# Prey Preferences of the Snow Leopard (*Panthera uncia*): Regional Diet Specificity Holds Global Significance for Conservation

**DOI:** 10.1371/journal.pone.0088349

**Published:** 2014-02-12

**Authors:** Salvador Lyngdoh, Shivam Shrotriya, Surendra P. Goyal, Hayley Clements, Matthew W. Hayward, Bilal Habib

**Affiliations:** 1 Department of Animal Ecology and Conservation Biology, Wildlife Institute of India, Chandrabani, Dehradun, Uttarakhand, India; 2 Nelson Mandela Metropolitan University, Port Elizabeth, South Africa; 3 College of Natural Sciences, Bangor University, Bangor, Gwynedd, United Kingdom; 4 Australian Wildlife Conservancy, Nichols Point, Australia; 5 Centre for African Conservation Ecology, Nelson Mandela Metropolitan University, Port Elizabeth, South Africa; Institut Pluridisciplinaire Hubert Curien, France

## Abstract

The endangered snow leopard is a large felid that is distributed over 1.83 million km^2^ globally. Throughout its range it relies on a limited number of prey species in some of the most inhospitable landscapes on the planet where high rates of human persecution exist for both predator and prey. We reviewed 14 published and 11 unpublished studies pertaining to snow leopard diet throughout its range. We calculated prey consumption in terms of frequency of occurrence and biomass consumed based on 1696 analysed scats from throughout the snow leopard's range. Prey biomass consumed was calculated based on the Ackerman's linear correction factor. We identified four distinct physiographic and snow leopard prey type zones, using cluster analysis that had unique prey assemblages and had key prey characteristics which supported snow leopard occurrence there. Levin's index showed the snow leopard had a specialized dietary niche breadth. The main prey of the snow leopard were Siberian ibex (*Capra sibrica*), blue sheep (*Pseudois nayaur*), Himalayan tahr (*Hemitragus jemlahicus*), argali (*Ovis ammon*) and marmots (*Marmota* spp). The significantly preferred prey species of snow leopard weighed 55±5 kg, while the preferred prey weight range of snow leopard was 36–76 kg with a significant preference for Siberian ibex and blue sheep. Our meta-analysis identified critical dietary resources for snow leopards throughout their distribution and illustrates the importance of understanding regional variation in species ecology; particularly prey species that have global implications for conservation.

## Introduction

Apex predators are often considered flagship species for conserving large landscapes due to their charisma and their dominant roles in shaping ecosystem functioning [1]–[4]. The snow leopard (*Panthera uncia*) is an icon for conservation in the mountain regions of Asia [5]. As a top-order predator, its presence and survival is also an indicator of intact, “healthy” eco-region [6]. The snow leopard is listed as endangered by the IUCN [7], and its abundance is declining across much of its present range. It is estimated that there are not more than 6500 mature individuals globally [7].

Conservation of key prey species is crucial for the survival of any large predator as changes in preferred prey abundance could alter its population status [8]–[10]. One of major reasons for the estimated 20% snow leopard population decline in the last two decades is a reduction in prey resource base [7]. Most of the snow leopard range overlaps with areas that have been overstocked with domestic ungulates [11]–[13]. In these areas, there has been a decline in wild prey availability [7], [14] due to competition for resources with domestic ungulates. In some areas, disease has further caused a rapid decline of wild prey [14]. The effects of such losses contribute to direct decline of snow leopards, as carrying capacity diminishes, and increased use of domestic livestock by snow leopards, elevating conflict and retaliatory killing by pastoralists [16]–[19]. Livestock depredation in such cases can also be substantial, varying from 2% to >10% [14], [16].

Snow leopards are elusive predators whose key habitats are alpine regions within altitudes of 900–4500 m [14], [19]. As they are difficult to observe and follow, there is a dearth of published information on snow leopard diet, as well as other aspects of its ecology, in comparison to other charismatic large carnivores [17], [18]. Hence fecal or scat analysis and diet profiles that offer insights into the predatory behavior of a species, also generate reliable assessments of levels of conflict due to livestock depredation if present [20]–[22].

A single snow leopard requires 1.5 kg of meat per day [23]. In general, their most commonly taken prey at individual sites consists of wild sheep and goats (blue sheep *Pseudois nayaur*, Siberian ibex *Capra sibirica*, markhor *Capra falconeri* and argali *Ovis ammon*), but their diet can also include pikas, hares, and game birds (chukar partridge *Alectoris chukar* and snowcock *Tetraogallus sp.*) [14]. In predation ecology, searching and pursuing time for prey are key factors which influence foraging strategies. Thus, a predator choses a foraging area and prey species, so as to minimize the sum of these two factors [15]. Studies have shown the local dietary requirements of the snow leopard; however these studies describe site-specific food habits of the species that are heavily influenced by available prey, hence an overview of food habits throughout the snow leopard's distribution is timely to identify the preferences in the snow leopard's diet with respect to prey type and size across varied landscapes.

We synthesized the available information of snow leopard diet from different regions and tested for regional differences in diet. Globally we compared (i) which large prey species are preferred/avoided and hence are crucial for the survival of the snow leopard? and (ii) whether there are separate zones, based alongside physiography and prey composition, where snow leopard ecology differs and hence may require unique conservation management strategies and (iii) what are the implications of this information for a global perspective in conservation?

## Materials and Methods

### Data compilation

Literature pertaining to the diet of snow leopard was accessed and reviewed. We searched relevant literature from Google Scholar and Web of Science, and grey literature such as dissertations and reports on snow leopard. A total of 25 (14 peer reviewed and 11 grey literature) studies were found on snow leopard diet that analyzed 1696 scats (Table 1). We obtained data from grey literature because these raw data were derived from standard, widely used analysis methods (scat analysis) and we made no use of the other methods, conclusions or inferences drawn within those reports (which are generally addressed in the peer-review process rather than the raw data provided robust methods are used), and we believe this is an appropriate use of grey literature. Furthermore, these studies are unlikely to bias our prey preference results because for a species to be significantly preferred or avoided several studies have to yield similar results [24]. Continuous observations are widely regarded as the superior method of ascertaining the diet of a large predator [25]; however, these are extremely difficult with such secretive and elusive predators as the snow leopard and so all studies relied on scat analysis.

**Table 1:** Sites and source of data used for this study.

S.No.	Sites	Source	Sample size	Zone
1	Balistan, Pakistan	Anwar *et al.* 2011	49	II
2	Kargil & Drass, India	Maheshwari & Sharma. 2010	9	II
3	Uttarakhand, Himachal India	Maheshwari & Sharma 2010	9	II
4	Kibber, India	Bagchi & Mishra 2006	44	II
5	Pin Valley, India	Bagchi & Mishra 2006	51	II
6	Ladakh, India	Chundawat and Rawat 1994	173	II
7	Northern, Nepal	Wegge, Shrestha & Flagstad 2012	41	I
8	Shey Phoksundo, Nepal	Devkota 2010	40	I
9	Dhorpatan, Nepal	Aryal 2009	23	I
10	Sagarmatha N. P, Nepal	Lovari *et al.* 2009	40	I
11	Sagarmatha N. P, Nepal	Lovari *et al.* 2009	66	I
12	KBR, Sikkim, India	Sathyakumar *et al.* 2009	117	I
13	Sagarmatha N. P, Nepal	Shrestha 2008	120	I
14	Langu Valley, Nepal	Jackson, 1996	78	I
15	Manang, Nepal	Oli *et al.* 1994	213	I
16	Taxkorgan, Xinjiang, China	Jun 2012	18	II
17	Wakhan, Afghanistan	Habib 2008	94	II
18	Chitral, Pakistan	Khatoon 2010	56	II
19	Kunlun Qinghai, China	Schaller 1988	13	III
20	Anyemaquen Qinghai, China	Schaller 1988	20	III
21	Zadoi Qinghai, China	Schaller 1988	36	III
22	Yushu Qinghai, China	Schaller 1988	46	III
23	Shule Nanshan Qinghai, China	Schaller 1988	91	III
24	South Gobi, Mongolia	Shehzad *et al.* 2012	81	IV
25	Uvs & South Gobi, Mongolia	Lhagvasuren & Munkhtsog 2000	168	IV

25 studies were referred spanning four zones in the snow leopard's distribution [18], [ 54]–[63].

**Figure 1:**
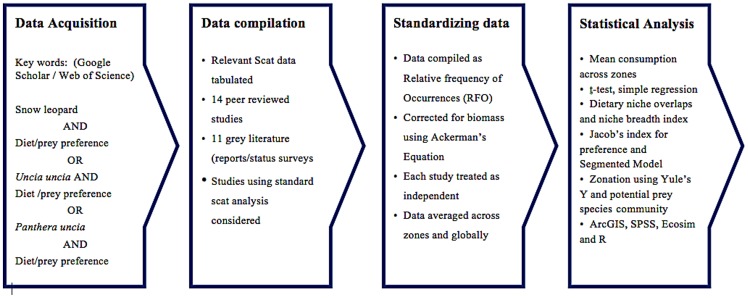
Flow chart of process employed for the current study.

We identified studies from 18 different areas in 6 countries describing the diet of the snow leopard, which included some measure of prey abundance (either actual or relative; Table 1; Fig. 1). All the studies were compiled and Frequency of Occurrence (hence forth FO) of *ith* prey item from the total occurrence of all prey items in scats was totalled. FO of prey items in scats for each prey species was multiplied with a conventional linear function used for cougars [29], which are similar in body mass to snow leopards and so are likely to have similar gut passage rates, to correct for biomass consumed per scat produced by multiplying average weight of prey i.e.[1.98+(0.035×Q1_kg_)]. This quantity gives the biomass consumed per unit scat for a species of prey. Total biomass consumed (Q2) can be calculated by (FO×Q1 = Q2). Each site within a study (i.e if more than one site) reviewed was considered as an independent observation. We calculated biomass consumed site wise and averaged them across all sites. Means and standard errors were calculated likewise. Total relative biomass contribution was represented as percentage for all species. We used three-quarters of the mean adult female body mass of prey species to take account of calves and sub-adults eaten following Schaller [30], and we continue its use here to allow comparison between studies. Prey weights were taken from Nowak [31]. Relative Frequency of Occurrence (RFO), which is (FO_ith species_/number of scats)×100, was used to compare prey consumption.

### Calculation of mean frequency of occurrence of prey items

Data were tabulated as relative (number of occurrences of each food item/total number of occurrences of all food items *100) frequencies of occurrence of each prey species. We used relative frequencies of all studies for overall comparisons. Using relative frequencies represented as percentages avoids the ambiguity of over-representation and standardizes the prey item occurrences [32]. These were averaged and then used to calculate snow leopard prey species consumption globally. Prey with body mass >40 kg were considered as large, prey with body mass <10 kg were placed in a small category and prey with body mass >10 and <40 were placed in the medium category. All statistical procedures were carried in SPSS 15.0 (Version 15.0. Chicago, SPSS Inc).

### Identification of unique conservation zones

We examined potential prey available in the 25 studies referred. Data of available prey were arranged in a 1-0 matrix and tested using Yule's Y Co-efficient of colligation in a cluster analysis to group the study area into unique zones based on prey communities. This is a function of the cross-product ratio for a 2×2 table and has a range of −1 to +1 [33]. Predation behavior of the snow leopard was examined with respect to the clusters formed from the above matrix.

### Diet specificity between zones

To determine the degree of dietary overlap between zones we used Pianka's dietary niche overlap index in Ecosim 7 (Acquired Intelligence Inc. Kesey-Bear, Pinyon Publishing 2011) [34]. We calculated cumulative Shannon diversity of prey items in each zone. Relative frequency of occurrence of prey species items in scats from unique zones identified were used in null model simulations of Pianka's dietary niche breadth, with relaxed and zero states retained [34]. We used Levin's index for computing dietary niche overlap between studies and averaged them for each zone [35]. Differences in relative proportion prey item of diet with respect to weight classes was tested using F-test.

### Prey preferences of snow leopard

We determined snow leopard prey preferences following the methods used to determine the prey preferences of other large predators [26]–[28]. Jacobs' index was used to determine the prey selectivity of snow leopards using the formula:
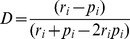
where, *r*_i_ is the proportion of species *i* among the total kills at a site and *p*_i_ is the proportion of species *i* in the available prey community [37]. The resulting values range from +1 (maximum preference) to −1 (maximum avoidance) [36]. The mean Jacobs' index value for each prey species across studies was calculated (±1 standard error (S.E.) wherever the mean is shown), and these values were tested for significant preference or avoidance using *t*-tests against an expected value of 0 as the data were normal. We used a segmented model of prey weight versus prey preference (Jacobs index values) to objectively quantify the weight ranges of prey preferred, prey killed relative to their abundance, and prey avoided following Clements *et al.* [38].

## Results

### Identification of unique snow leopard zones

**Figure 2:**
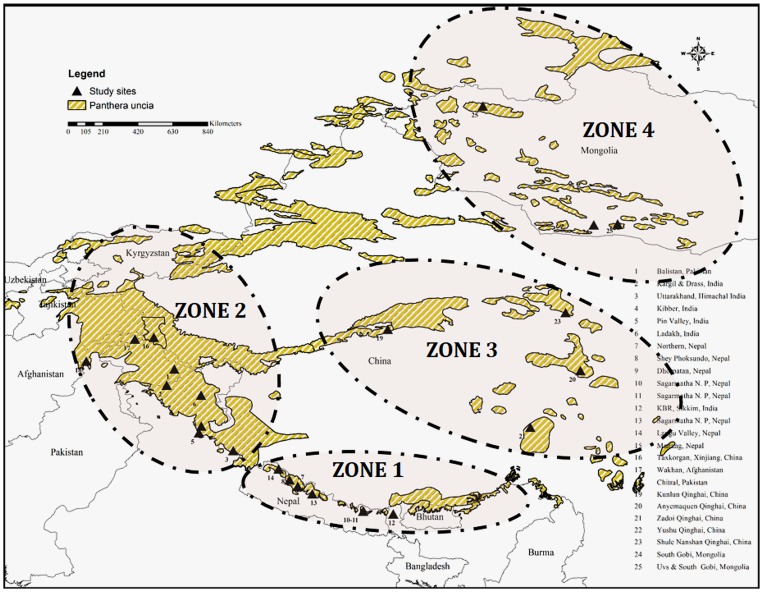
Location of 25 study sites and four physiographic and prey type zones for snow leopard conservation. (Reproduced from IUCN 2012. *IUCN Red List of Threatened Species.* *Version 2012.1*. http://www.iucnredlist.org. Downloaded on 22-02-2013).

**Figure 3:**
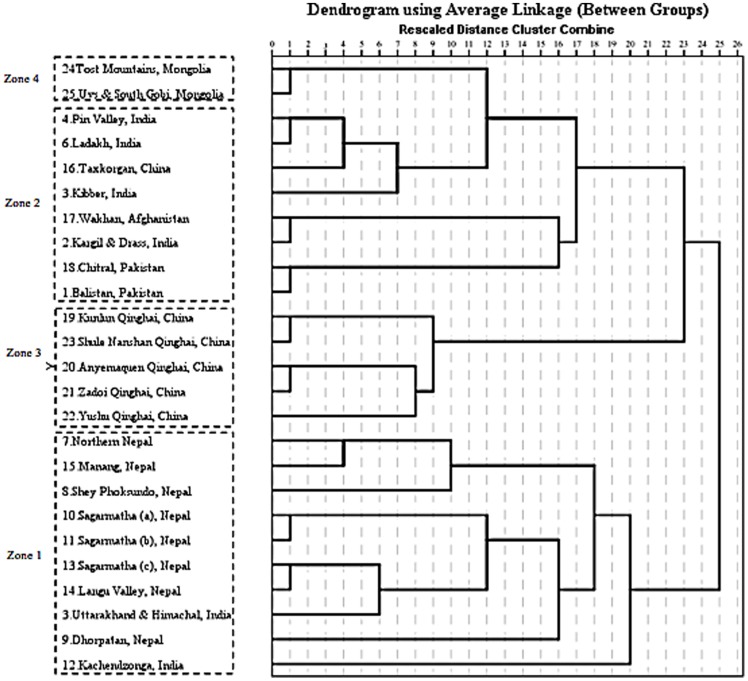
Zonation dividing the four zones using Yule ‘Y’ colligation co-efficient. Bases on potential prey available in study areas of referred. Clusters formed in to 4 zones based on potential prey (1-0 matrix) available in the study site. Nearest linkage are close to 0 farthest are towards 25. On average groups were at 25 and 20 (Zone 1), 9 (Zone 3), 23, 17 (Zone 2), 8 and 1 (Zone 4). Zone 4 separated out as a sub cluster but due to it's physiographical nature this zone is treated as distinct.

We identified four distinct zones of the snow leopard and studied their predation behavior in these zones. All the four zones are geographically distinct from each other (Fig. 2 & 3). Zone 1 included the Indian Himalayas from Himachal Pradesh and Uttarakhand towards Nepal, Sikkim and Arunachal Pradesh. Zone 2 included the Trans-Himalayan parts west to Uttarakhand, Pakistan and north-west Afghanistan. Zone 3 comprised of Kunlun and Qinghai central regions of China. The Zone 4 included the Mongolian regions of Altai, south Gobi and Uvs. In Zone 1, wild prey species included blue sheep and Siberian ibex, Zone 2 prey include ibex, argali and markhor, Zone 3 prey include blue sheep and marmot (*Marmorata* spp), while Zone 4 prey species include Siberian ibex, white lipped deer (*Prezewalskium albirostris*) and Siberian roe deer (*Capreolus pygargus*).

### Diet diversity in scats among zones

**Figure 4:**
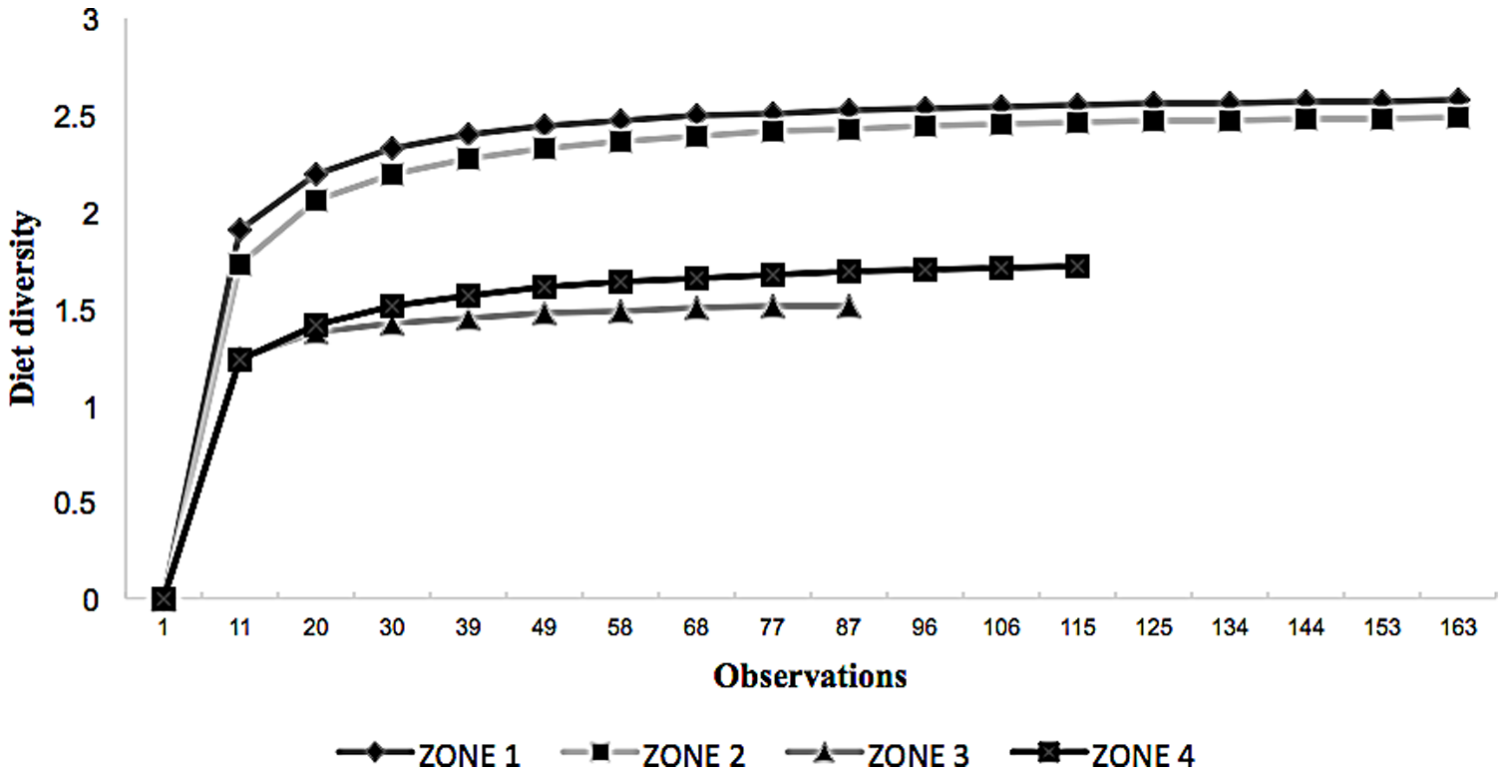
Diet diversity in different zones. Cumulative observations of FOO of prey in scats were used from each zone. Diet diversity is represented by Shannon index.

Mean dietary diversity was highest in Zone 1 (2.57), followed by Zone 2 (2.49), then Zone 3 (1.51) and Zone 4 (1.72). Species diversity curves revealed that scat sample sizes more or less stabilized for all zones (Fig. 4) indicating that they were adequately sampled.

Pianka's dietary niche overlap ranged between 5–50% between all the zones. Dietary niche overlap showed that Zone 1 had an overlap of 33.27% with Zone 2, 48.70% overlap with Zone 3 and 11.31% overlap with Zone 4. Zone 2 and Zone 3 had a dietary overlap of 40.50% while there was a 50.60% overlap between Zone 2 and Zone 4. Zone 3 and Zone 4 had a dietary overlap of 4.58%. The mean overlap across zones was 31.52%. Dietary niche overlap was not significant among the zones (i.e observed mean was 31.52%±0.81% whereas the simulated mean was 24.75±0.2% (p>0.05). Similarly the mean of observed variances (0.03%) was not significantly different from the null model (0.02%, p>0.05).

**Figure 5:**
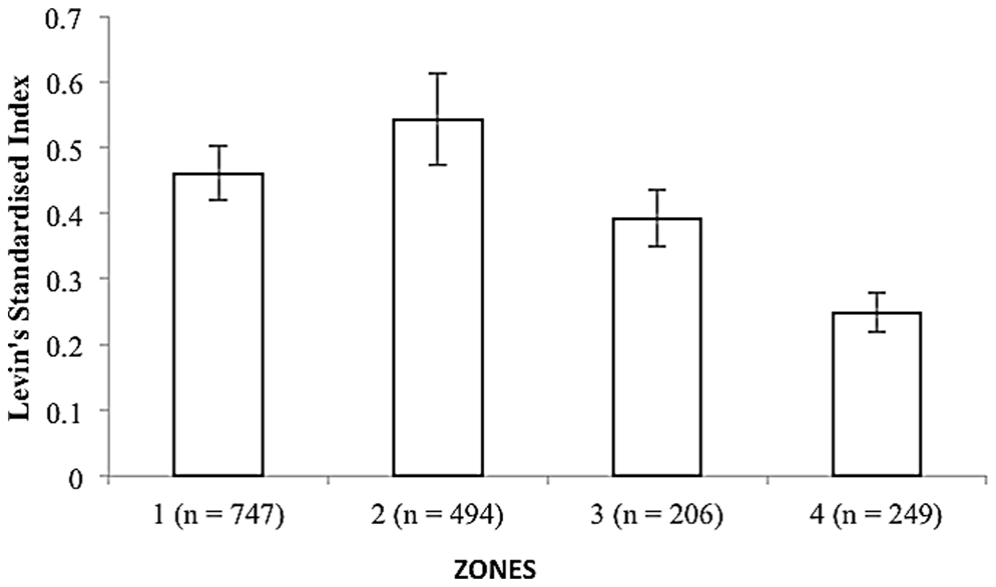
Levin's standardized Index showing dietary niche breadth between the zones. Error bars represent (±1) S.E from the mean.

Levin's dietary niche breadth index (standardised) showed similar trends as in other indices for different zones (Fig. 5). Zone 1 showed a niche breadth of 0.46±0.04, Zone 2, 0.54±0.07 had a high niche breadth.

### Prey consumption

**Figure 6:**
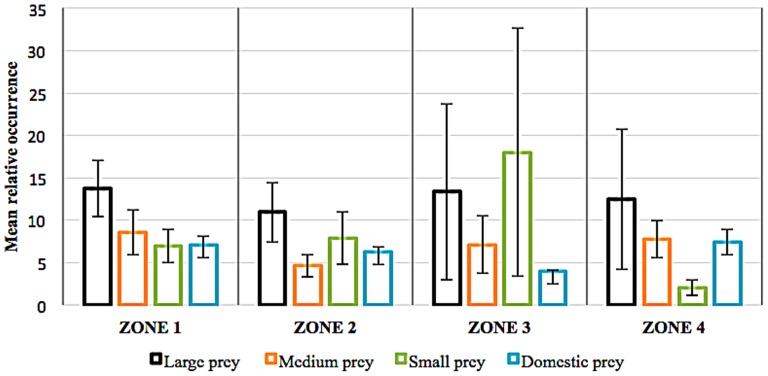
Mean Relative Occurrence of prey species in the diet of snow leopard across different zones. Mean relative occurrences were calculated as averages of relative occurrences of prey item in scat in each zone (large prey >40 kg, medium prey >10 and <40 kg, small prey <10 kg).

**Figure 7:**
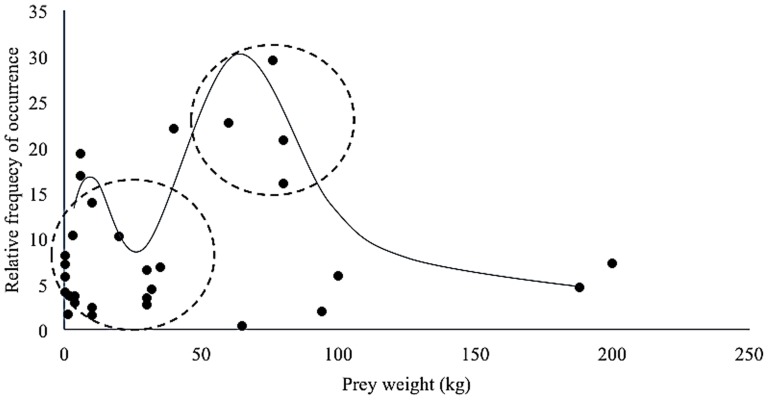
Prey weight and relative occurrence of prey in snow leopard scat. Prey weights used were ¾ the body mass of average adult female of each prey species. Double hump indicates that snow leopard feeds primarily on large prey but may shift to small-bodied prey sub-optimally.

We found that the top four species detected in snow leopard scats were Siberian ibex (29.54±12.40% RFO), Himalayan tahr (22.62±7.21%), blue sheep (21.97±9.37%) and argali (20.8±12.20%). Small prey also occurred frequently in snow leopard scats: marmots (19.31%±9.86%), civets (16.80%) and rodents (6.34±1.80%). Among medium-sized wild prey, the musk deer was killed by the snow leopard (13.00±0.11%) relatively frequently. Domestic livestock contributed to less than 8% across all the four Zones (Fig. 6 and 7).

Proportion of large, medium and small prey in snow leopard scats were constant (Fig. 6) across the zones. (F_2,2_ = 0.06, p = 0.98). The large prey that contributed most in snow leopard diet in Zone 1 were blue sheep (31.07±4.22%) and Himalayan tahr (22.62±7.21%). In Zone 2, prey that occurred most frequently in snow leopard's diet was argali (33.00%, one study), Siberian ibex (18.37±5.87%) and blue sheep (13.96±5.76%). Zone 3 showed that blue sheep (34±3.66%), and marmots (47.22±5.10%) were predominately consumed. Principal prey species that occurred frequently in Zone 4 were Siberian ibex (53.53±16.87%) along with domestic goats (10.35±6.95%) and sheep (9.44±6.94%). Civet (16.80%) in Zone 2 was reported by one study. Rodent species contributed >10% of the diet in Zone 1 and Zone 2. Mean relative domestic livestock consumption was high in Zone 1 (i.e. 7.04±1.05%) and lowest in Zone 3 (i.e. 3.93%±0.18%; Fig. 6).

### Prey preferences

Siberian ibex, Himalayan tahr, goats, sheep, rodents and horses were killed by snow leopards wherever they occurred. Cattle/yak were the prey killed by snow leopards at most study sites, but not invariably, as were blue sheep Blue sheep, cattle and goats were the most abundant prey species available in the prey communities studied, while blue sheep, goats and Himalayan tahr were the most frequently preyed upon (Table 2).

**Table 2:** Overall frequency of occurrence and biomass consumed by snow leopard (*Panthera uncia*).

Prey Species	Weight used (kg)	Biomass consumed per scat (kg)	Average Biomass consumed (kg)	SE (kg)	Percentage (%) biomass contribution	Na	Np	Jacobs' index	Abundance	Prey
Argali *(ovis ammon)*	80	4.78	90.79	64.20	12.71	6	2	−0.75±0.25	0.14±0.10	0.02±0.02
Siberian Ibex *(Capra sibrica)*	76	4.64	90.66	30.22	12.69	6	6	0.48±0.15	0.16±0.06	0.17±0.07
Cattle and Yak *(Bos spp)*	200	8.98	68.62	16.64	9.60	13	11	−0.41±0.16	0.24±0.09	0.14±0.02
Blue Sheep *(Pseudois nayaur)*	40	3.38	67.49	17.43	9.45	10	9	0.43±0.17	0.54±0.11	0.45±0.12
Himalayan tahr *(Hemitragus jemlahicus)*	50	3.73	57.04	23.29	7.98	5	5	0.32±0.29	0.11±0.05	0.24±0.08
Domestic Dog *(C.l.familairis)*	20	2.68	32.93	32.93	4.61	1	1			
Marmots *(Marmorata spp)*	6	2.19	30.78	8.54	4.31	6	5	−0.12±0.23	0.14±0.04	0.08±0.02
Horse *(Equus ferus caballus)*	188	8.56	30.75	10.25	4.30	7	7	−0.31±0.20	0.04±0.01	0.08±0.02
Royale's Vole *(Alticola royle)*	0.3	1.99	24.49	24.49	3.43	1	1			
Pika *(Ochotona spp)*	0.3	1.99	23.51	8.31	3.29	6	5	−0.16±0.25	0.13±0.07	0.10±0.06
Musk Deer *(Moschus leucogaster)*	10	2.33	22.51	10.07	3.15	7	5	−0.03±0.37	0.03±0.03	0.17±0.06
Palm Civet *(Paradoxurus hermaphroditus)*	6	2.19	20.60	20.60	2.88	1	1			
*Wild Pig (Sus scrofa)*	80	4.78	17.65	17.65	2.47	1	1			
Goitered Gazelle *(Gazella subgutturosa)*	30	3.03	17.35	17.35	2.43	4	0	−1±0	0.01±0.01	0
Weasel *(Mustela spp)*	2	2.05	15.76	15.76	2.21	1	1			
Marten *(Martes flavigula)*	4	2.12	13.04	13.04	1.83	1	1			
Goat *(Capra aegagrus hircus)*	35	3.21	12.55	2.96	1.76	7	7	−0.44±0.13	0.21±0.09	0.26±0.06
Sheep *(Ovis aries)*	30	3.03	12.42	3.01	1.74	6	6	−0.21±0.22	0.12±0.05	0.20±0.05
Rodents *(Rodentia spp)*	0.3	1.99	12.19	3.52	1.71	5	5	0.05±0.28	0.10±0.04	0.06±0.01
Donkey *(Equus africanus asinus)*	100	5.48	10.46	5.23	1.46	3	0	−1±0	0.01±0.01	0
White lipped deer *(Prewalskium albirostris)*	94	5.27	10.24	5.91	1.43	4	2	−0.56±0.38	0.06±0.05	0.02±0.01
Markhor *(Capra falconeri)*	80	4.78	7.29	5.16	1.02	2	2	0.07±0.59	0.04±0.02	0.12±0.04
Hare *(Lepus spp)*	4	2.12	6.94	2.31	0.97	5	4	0.20±0.24	0.08±0.04	0.07±0.05
Birds	0.3	1.99	5.29	1.41	0.74					
Red Fox *(Vulpes vulpes)*	10	2.33	3.36	2.37	0.47	1	1			
Rhesus Macaque *(Macaca mulata)*	10	2.33	3.13	3.13	0.44	1	1			
Ladakh Urial *(Ovis orientalis)*	65	4.26	2.94	2.94	0.41	1	1			
Goral *(Naemorhedus goral)*	30	3.03	1.86	1.86	0.26	1	1			
Squirrel *(Sciuridae spp)*	1.5	2.03	1.82	1.82	0.25	1	1			

Frequency of occurrence was calculated as relative frequency of occurrence. Ackerman's correction factor was used for estimating biomass consumed. N_a_ refers to studies where a species occurred in the prey community and N_p_ refers to those studies where the species was killed by snow leopards. Abundance and Prey refer to the data used to calculate Jacob's index.

**Figure 8:**
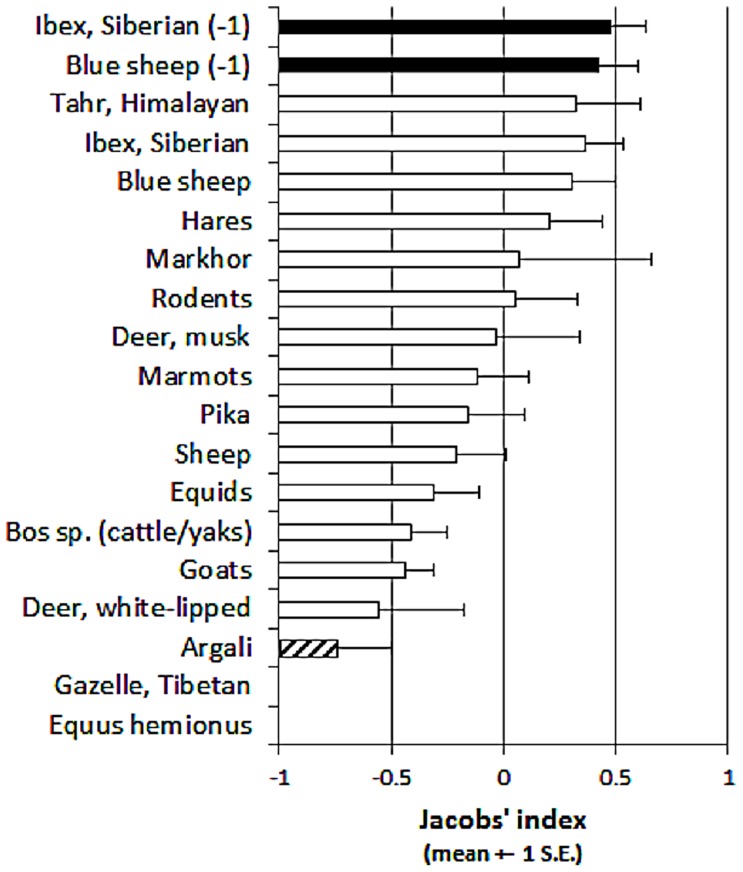
Mean Jacobs' index values (±1 S.E.) for prey species of the snow leopard at two or more sites. Black illustrates significantly preferred prey, open bars represent species killed in proportion to their availability and stippled bars (or no bar) indicate significantly avoided prey species. As described in the text, we analysed the data of Siberian ibex and blue sheep twice to remove one outlying result for each.

**Figure 9:**
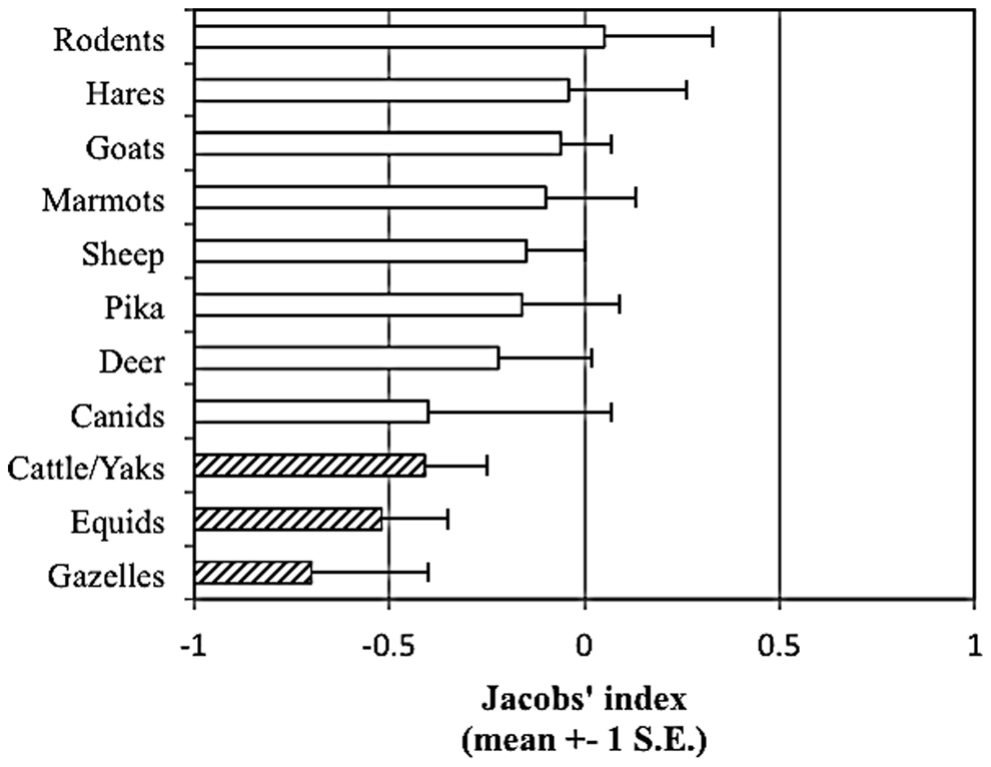
Prey preferences of taxonomic groups of snow leopard prey items. Black illustrates significantly preferred prey, open bars represent species killed in proportion to their availability and stippled bars (or no bar) indicate significantly avoided prey species.

When all data were included, snow leopards exhibited no prey preferences but significantly avoided Asiatic wild asses (*Equus hemionus*) and Tibetan gazelles (*Procapra picticaudata*), which were never preyed upon) and argali (*t*_5_ = −3.05, *p* 0.028; Fig. 8). However excluding one outlying sample, led to Siberian ibex and blue sheep becoming significantly preferred (ibex *t*_5_ = 2.598, *p* = 0.048; blue sheep *t*_8_ = 2.478, *p* = 0.038). These preferences are not driven by preferences for entire taxonomic groups (Fig. 9).

**Figure 10:**
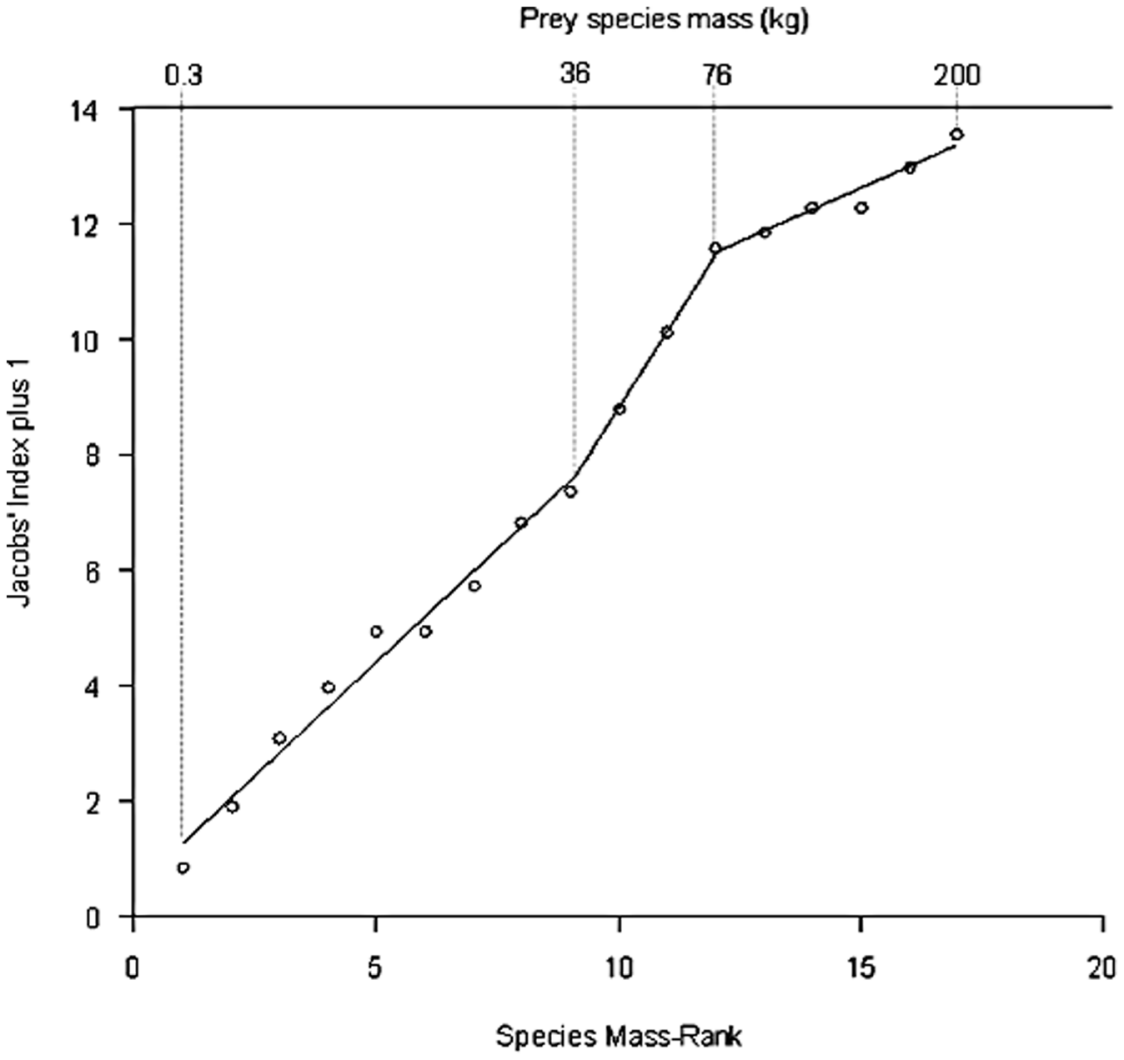
Segmented model of the relationship between the mass-rank of each prey species and the corresponding cumulative Jacobs' Index +1 value for snow leopard. Actual prey species' masses, which correspond to the lowest, break-point, and highest prey mass-ranks are indicated.

The body mass of significantly preferred prey was 50±5 kg according to Jacob's index values calculated. There are two significant changes in the relationship between prey species mass-rank and snow leopard prey preference (AIC = 9.13, n = 17). These occur at a prey species mass-rank of 8.4 and 11.7, corresponding to a prey species mass of 36 kg and 76 kg, respectively (Fig. 10). Prey species weighing 40 kg or less are killed relative to their abundance (*D*. = −0.06±0.17, t = −0.38, d.f. = 15, p = 0.71), prey species weighing between 36 kg and 76 kg are preferred (*D*. = 0.35±0.16, t = 2.22, d.f. = 16, p = 0.04) and prey species weighing more than 76 kg are avoided (*D*. = −0.44±0.13, t = −3.29, d.f. = 16, p<0.01).

### Biomass consumption

From Ackerman's correction, the overall relative biomass contribution was high for Argali (12.71%) Siberian ibex (12.69%), cattle and yak (9.60%) blue sheep (9.45%) and Himalayan tahr (7.98%). These prey contributed to 52.43% of the total biomass contribution in snow leopard diet globally. Small prey species that contributed in larger numbers to biomass of the snow leopard diet were marmots (4.31%), voles (3.43%) and pikas (3.39%). Small prey, including pikas and voles contributed about 16.90% of the total prey biomass consumed globally by snow leopard. Domestic cattle and yak also contributed to high proportions to the diet of the snow leopard (Table 2). Globally, domestic livestock including cattle, yak, domestic dogs, horses, donkey, sheep and goat constitute 23.47% to the total prey biomass consumed by snow leopard.

## Discussion

The snow leopard has specialized needs, having evolved to prey primarily on large-bodied prey (36–76 kg). Snow leopards are behaviorally and morphologically adapted to become successful predators in the harsh, resource-limited terrain they inhabit that probably limits movement of females across different habitats and between zones [6]. Each zone is unique with respect to its prey composition as well as physiography. All the four zones separated satisfactorily, however Zone 2 and Zone 4 were treated as separate zones due to their geographic separation as well as prey diversity. A larger sample size from Zone 4 would have revealed distinct prey composition. Within these zones there may still exist crucial units where source populations of snow leopards thrive. Our results highlight the fact that prey preference of the snow leopard has not evolved on broader groups (i.e. sheep or goats), but is species specific (Fig. 9).

### Prey consumption patterns

Prey item diversity in scats increased at lower latitudes [38], [39] in carnivores. The studies we used accounted for the maximum number of prey species that may have been encountered from scat analysis since the cumulative species richness graphs showed asymptotes for all zones.Levin's standardised niche index indicated that the snow leopard was particularly specialized in Zone 3 and Zone 4. This is counter-intuitive considering optimal foraging theory predicts animals should be less specialized where resources are scarce [40], [41], [42]. Rather, this reflects the lack of prey available in these zones. All zones showed some degree of dietary overlaps ranging from 5–50% (mean 31.52±0.81%) possibly due to contribution of livestock mainly in all the zones.

### Predatory needs

There cannot be a globally single important prey for the snow leopard's survival, due to the localized distribution of snow leopard prey species compared to snow leopard distribution each zone supports a unique prey base of species which are crucial for the snow leopard. However, availability of prey weighing between 36 kg and 76 kg is important in all zones. Main preferred prey species are blue sheep and Siberian ibex. Himalayan tahr also constitutes an important prey in Zone 1 (22. 62±7.21). The results are indicative of evolutionary association of snow leopard and associated prey species, akin to the tight co-evolutionary relationship between tigers (*Panthera tigris*) and large cervids [27]. Fortunately, endangered prey like the Ladakh urial (*Ovis vignei*) and the markhor (*Capra falconeri*) are unimportant for snow leopards (<2.00% and <6.00% of overall diet respectively) reflecting the protection that inherent rarity in the prey community affords prey from the evolution of preferential predation [41], [43].

It is evident that large prey constituted the major proportion of the snow leopard's diet since in all the four zones these prey were consumed in similar proportions. Medium-sized prey constituted a minor part of the snow leopard's diet and these were either sheep or goats (6.68%, Fig. 6). Zone 3 showed much variability in mean relative occurrences of prey as sample sizes were low. This percentage is contrary to popular belief that snow leopard primarily prey on livestock [16]. Locally, however situations may vary [16], [18] and in some sites nearly 40% of the snow leopard's diet is comprised of livestock. The consumption of livestock by snow leopard may be driven largely by overabundance in Zone 1and Zone 2 as these rangelands are traditionally overstocked.

A large predator will maximize its prey choice based on optimal foraging theory and follow a normal distribution pattern, hence larger prey requiring a relatively balanced energy expense to catch safely will be chosen over smaller prey too little to bother with [24], [26], [44], [45]. Therefore, like other large, solitary predators, the snow leopard is expected to kill prey species with similar body weights to itself [45] or similar to a common leopard's dietary requirements, which is an optimum of 24 kg (10–40 kg) [27]. Likewise, we found snow leopard significantly preferred prey weighing 50±5 kg. This leads to a predator to preferred prey ratio of 1∶1.29 based on an adult female snow leopard body mass of 38.5 kg (http://www.sandiegozoo.org). Body weight versus frequency of relative occurrence shows a skewed pattern to the right and a double hump (Fig. 8) suggesting that snow leopards opportunistically kill smaller prey but prefer large prey within a 36–76 kg weight class (Fig. 10). As in the case with large solitary predators too large a prey i.e beyond 76 kg is difficult and risky to catch and is generally avoided [48].

Zone type along with weight class influences the relative consumption of prey. Like other large, solitary predators, tigers (*Panthera tigris*) and leopards (*P. pardus*), snow leopards may need to be more selective about the habitat zone they forage in and the weight class of prey they target in order to maximize hunting success and reduce injury risk [47], [49]. Indeed, it is likely that snow leopards, like lions (*P. leo*) reinforce their prey preferences throughout the predatory behavioural sequence by preferentially foraging in habitats where preferred prey occur, and by more frequently initiating hunts of preferred prey [50], [51]. Predatory skills acquired by cubs through their mother may have led them to be more successful predators in accordance with physiographic characteristics of each zone. This also may have exclusively restricted movements of females from one zone to another thus expressing observed unique haplotype in cytochrome gene analysis at a global level (Goyal *et al.*, unpublished data)

In many studies of diets of large predators in tropical regions, obligate carnivores may consume sub-optimal, smaller prey due to an absence of principal large prey [46], [50], [51]. It is noteworthy that in Zone 3, marmots have a high conservation value since they constitute an important part of the snow leopard diet there. From all these observations, it is clear that the snow leopard is a specialized predator relying on large wild prey primarily but will forage optimally by being more generalist when preferred prey is scarce. This is reinforced by the total of 30 prey species identified in the diet of the snow leopard.

### Biomass consumed

Siberian ibex, Himalayan tahr, blue sheep and argali were the most important prey based on their relative occurrence in the scats as they accounted for nearly 42.83% of the biomass consumed based on Ackerman's equation. Nearly 76.52% of the snow leopard's dietary requirements were met from wild prey alone [50]. Domestic livestock contributed to less than 23.48% of the overall diet of the snow leopard, even though they were invariably killed by snow leopards where they coexisted (Table 2). However, cattle and yak were among the four most consumed prey of the snow leopard in terms of biomass.

### Future implications of the study in the snow leopard's range

Our findings have revealed three key large prey species that are cornerstone of the conservation of the snow leopard globally i.e blue sheep, ibex and Himalayan tahr. These prey species occur throughout the snow leopard range and hence are vital resource as its prey. Another, salient piece of information derived is that prey species of an optimal weight class category (36–76 kg) is generally preferred by the snow leopard. Thus conserving prey within this weight category may guide management of wild prey populations in the future. Furthermore, if conservation of the snow leopard is to be a long term investment [52], focal research is needed on these preferred prey populations, as well as reducing livestock depredation rates and maintaining the integrity of such landscapes. Our study delineates such important areas (i.e., at a regional scale). Our findings show that globally, each zone has a unique potential in supporting ungulate prey biomass and directs snow leopard presence. Specific conservation units within these zones may be ultimate refuges for source populations. Having stated this, maintaining connectivity between such refuges and zones will add as an insurance against loss of viability in snow leopard populations. All zones have leopard-human conflicts due to livestock depredation (Table 2, Fig. 6), thus ensuring intactness of wild prey populations and controlling wildlife-human conflicts through better protection of livestock is imperative [23], [53]. Other prey such as argali and marmots play an important part as they support snow leopard populations in areas devoid of major preferred prey. Small prey also play an important role in the diet of the snow leopard in some regions and seasons, where a broader range of prey would be eaten by species in more resource-poor environments. Thus, prey abundance and distribution on a regional scale has global impacts in shaping a significant part of the snow leopard's ecological future and maintaining genetic diversity of this species across its range.
