# Correction: Prey Preferences of the Snow Leopard (*Panthera uncia*): Regional Diet Specificity Holds Global Significance for Conservation

**DOI:** 10.1371/journal.pone.0100071

**Published:** 2014-06-06

**Authors:** The PLOS ONE Staff

There is an error in the first column of Table 2.

Please view the correct Table 2 here:

**Table 2:** **Overall frequency of occurrence and biomass consumed by snow leopard (** ***Panthera uncia*** **).**

Prey Species	Weight used (kg)	Biomass consumed per scat (kg)	Average Biomass consumed (kg)	SE (kg)	Percentage (%) biomass contribution	Na	Np	Jacobs’ index	Abundance	Prey
*Argali (Ovis ammon)*	80	4.78	90.79	64.20	12.71	6	2	-0.75 ± 0.25	0.14 ± 0.10	0.02 ± 0.02
*Siberian Ibex (Capra sibirica)*	76	4.64	90.66	30.22	12.69	6	6	0.48 ± 0.15	0.16 ± 0.06	0.17 ± 0.07
*Cattle and Yak (Bos spp)*	200	8.98	68.62	16.64	9.60	13	11	-0.41 ± 0.16	0.24 ± 0.09	0.14 ± 0.02
*Blue Sheep (Pseudois nayaur)*	40	3.38	67.49	17.43	9.45	10	9	0.43 ± 0.17	0.54 ± 0.11	0.45 ± 0.12
*Himalayan tahr(Hemitragus jemlahicus)*	50	3.73	57.04	23.29	7.98	5	5	0.32 ± 0.29	0.11 ± 0.05	0.24 ± 0.08
*Domestic Dog (C.l.familairis)*	20	2.68	32.93	32.93	4.61	1	1			
*Marmots (Marmota spp)*	6	2.19	30.78	8.54	4.31	6	5	-0.12 ± 0.23	0.14 ± 0.04	0.08 ± 0.02
*Horse (Equus ferus caballus)*	188	8.56	30.75	10.25	4.30	7	7	-0.31 ± 0.20	0.04 ± 0.01	0.08 ± 0.02
*Royale's Vole (Ochotona roylei)*	0.3	1.99	24.49	24.49	3.43	1	1			
*Pika (Ochotona spp)*	0.3	1.99	23.51	8.31	3.29	6	5	-0.16 ± 0.25	0.13 ± 0.07	0.10 ± 0.06
*Musk Deer (Moschus leucogaster)*	10	2.33	22.51	10.07	3.15	7	5	-0.03 ± 0.37	0.03 ± 0.03	0.17 ± 0.06
*Palm Civet (Paradoxurus hermaphroditus)*	6	2.19	20.60	20.60	2.88	1	1			
*Wild Pig (Sus scrofa)*	80	4.78	17.65	17.65	2.47	1	1			
*Goitered Gazelle (Gazella subgutturosa)*	30	3.03	17.35	17.35	2.43	4	0	-1 ± 0	0.01 ± 0.01	0
*Weasel ( Mustela spp)*	2	2.05	15.76	15.76	2.21	1	1			
*Marten (Martes flavigula)*	4	2.12	13.04	13.04	1.83	1	1			
*Goat (Capra aegagrus hircus)*	35	3.21	12.55	2.96	1.76	7	7	-0.44 ± 0.13	0.21 ± 0.09	0.26 ± 0.06
*Sheep (Ovis aries)*	30	3.03	12.42	3.01	1.74	6	6	-0.21 ± 0.22	0.12 ± 0.05	0.20 ± 0.05
*Rodents (Rodentia spp)*	0.3	1.99	12.19	3.52	1.71	5	5	0.05 ± 0.28	0.10 ± 0.04	0.06 ± 0.01
*Donkey (Equus africanus asinus)*	100	5.48	10.46	5.23	1.46	3	0	-1 ± 0	0.01 ± 0.01	0
*White lipped deer (Przewalskium albirostris)*	94	5.27	10.24	5.91	1.43	4	2	-0.56 ± 0.38	0.06 ± 0.05	0.02 ± 0.01
*Markhor (Capra falconeri)*	80	4.78	7.29	5.16	1.02	2	2	0.07 ± 0.59	0.04 ± 0.02	0.12 ± 0.04
*Hare (Lepus spp)*	4	2.12	6.94	2.31	0.97	5	4	0.20 ± 0.24	0.08 ± 0.04	0.07 ± 0.05
*Birds*	0.3	1.99	5.29	1.41	0.74					
*Red Fox (Vulpes vulpes)*	10	2.33	3.36	2.37	0.47	1	1			
*Rhesus Macaque (Macaca mulata)*	10	2.33	3.13	3.13	0.44	1	1			
*Ladakh Urial (Ovis orientalis)*	65	4.26	2.94	2.94	0.41	1	1			
*Goral (Naemorhedus goral)*	30	3.03	1.86	1.86	0.26	1	1			
*Squirrel (Sciuridae spp)*	1.5	2.03	1.82	1.82	0.25	1	1			

Frequency of occurrence was calculated as relative frequency of occurrence. Ackerman’s correction factor was used for estimating biomass consumed. N_a_ refers to studies where a species occurred in the prey community and N_p_ refers to those studies where the species was killed by snow leopards. Abundance and Prey refer to the data used to calculate Jacob’s index.

Additionally, there is an error in the first sentence of the second paragraph of the sub-heading “Prey preferences” under the “Results” section. The correct sentence for this section is:

When all data were included, snow leopards exhibited no prey preferences but significantly avoided Tibetan wild ass (*Equus kiang*) and Tibetan gazelles (*Procapra picticaudata*), (which were never preyed upon) and argali (*t*_5_  =  −3.05, *p* 0.028; Fig. 8).

## References

[1] Lyngdoh S, Shrotriya S, Goyal SP, Clements H, Hayward MW, et al (2014) Prey Preferences of the Snow Leopard (Panthera uncia): Regional Diet Specificity Holds Global Significance for Conservation. PLoS ONE 9(2): e88349 doi:10.1371/journal.pone.0088349 24533080 10.1371/journal.pone.0088349 PMC3922817

